# Vitamin D in a Northern Canadian First Nation Population: Dietary Intake, Serum Concentrations and Functional Gene Polymorphisms

**DOI:** 10.1371/journal.pone.0049872

**Published:** 2012-11-21

**Authors:** Linda Larcombe, Neeloffer Mookherjee, Joyce Slater, Caroline Slivinski, Matthew Singer, Chris Whaley, Lizette Denechezhe, Sara Matyas, Emily Turner-Brannen, Peter Nickerson, Pamela Orr

**Affiliations:** 1 Faculty of Medicine, Department of Internal Medicine, University of Manitoba, Winnipeg, Manitoba, Canada; 2 Faculty of Medicine, Department of Medical Microbiology, University of Manitoba, Winnipeg, Manitoba, Canada; 3 Faculty of Medicine, Department of Community Health Sciences, University of Manitoba, Winnipeg, Manitoba, Canada; 4 Manitoba Centre for Proteomics and Systems Biology, University of Manitoba, Winnipeg, Manitoba, Canada; 5 Department of Human Nutritional Sciences, University of Manitoba, Winnipeg, Manitoba, Canada; 6 Northlands Denésuline First Nation, Lac Brochet, Manitoba, Canada; Brigham & Women’s Hospital, and Harvard Medical School, United States of America

## Abstract

The wide spectrum of vitamin D activity has focused attention on its potential role in the elevated burden of disease in a northern Canadian First Nations (Dené) cohort. Vitamin D insufficiency, and gene polymorphisms in the vitamin D receptor (VDR) and vitamin D binding protein (VDBP) have been implicated in susceptibility to infectious and chronic diseases. The objectives of this study were to determine the contribution of vitamin D from food, and measure the serum concentrations of 25-hydroxyvitamin D_3_ (25-OHD_3_) and VDBP in Dené participants. Single nucleotide polymorphisms (SNPs) associated with the dysregulation of the innate immune response were typed and counted. Potential correlations between the SNPs and serum concentrations of 25-OHD_3_ and VDBP were evaluated. Venous blood was collected in summer and winter over a one-year period and analyzed for 25-OHD_3_ and VDBP concentrations (N = 46). A questionnaire was administered to determine the amount of dietary vitamin D consumed. Sixty-one percent and 30% of the participants had 25-OHD_3_ serum concentrations <75 nmol/L in the winter and summer respectively. Mean vitamin D binding protein concentrations were within the normal range in the winter but below normal in the summer. VDBP and VDR gene polymorphisms affect the bioavailability and regulation of 25-OHD_3_. The Dené had a high frequency of the VDBP D432E-G allele (71%) and the Gc1 genotype (90%), associated with high concentrations of VDBP and a high binding affinity to 25-OHD_3_. The Dené had a high frequency of VDR Fok1-f allele (82%), which has been associated with a down-regulated Th1 immune response. VDBP and VDR polymorphisms, and low winter 25-OHD_3_ serum concentrations may be risk factors for infectious diseases and chronic conditions related to the dysregulation of the vitamin D pathway.

## Introduction

Vitamin D has a wide spectrum of activity including calcium and bone homeostasis, cardiovascular and immune system function, as well as skin, muscle and cell proliferation. The elevated burden of both infectious and non-infectious diseases borne by Canada’s Aboriginal (First Nations, Metis and Inuit) people has focused attention on the potential causal, preventive and/or therapeutic role, if any, of this vitamin [1]. Case reports of rickets, elevated fracture risk and low bone mineral density in First Nations and Inuit children and women suggest that vitamin D deficiency is not rare in these groups [2]–[4]. There are currently no published data on the gene-nutrient interaction with regards to vitamin D in Canadian northern First Nation populations.

Vitamin D is derived nutritionally from a limited number of foods. The primary source comes from the skin conversion of 7-dehydrocholesterol, induced by exposure to solar ultraviolet B (UVB) radiation. Vitamin D is converted in the liver to 25-hydroxyvitamin D_3_ (25-OHD_3_) and further hydroxylated in the kidney to 1,25-dihyroxyvitamin D_3_ (1,25(OH)_2_D_3_), the most active form of vitamin D_3_. Serum 25-OHD_3_ concentrations are used as the clinical measure of vitamin D status. In addition to the classical function of vitamin D on skeletal development, 1,25(OH)_2_D_3_ binds with VDRs found in many tissue types to regulate cell growth and maturation, stimulate insulin secretion, and modulate the function of activated T- and B-lymphocytes and macrophages [5].

Serum 25-OHD_3_ is transported to organs, tissues and cells by VDBP (also known as group-specific component, or Gc) which regulates the availability of serum vitamin D and its metabolites [6]. Circulating 25-OHD_3_ is bound to VDBP, enters macrophages, is converted to 1,25(OH)_2_D_3_ by mitochondrial CP27B, and then binds to the VDR in the cell. Once bound to VDR, 1,25(OH)_ 2_D_3_ mediates the induction of human cathelicidin, an immunomodulatory peptide contributing to antimicrobial activity against pathogens including *Mycobacterium tuberculosis* [7], [8]. In a recent *in vitro* study a single dose of vitamin D (2.5 mg) enhanced anti-mycobacterial immunity among tuberculosis contacts [9]. In a longitudinal study that tested the effects of vitamin D supplementation on *Mycobacterium tuberculosis*-induced innate immune response in the Dené we found that vitamin D supplementation did not enhance the innate immune response to *Mycobacterium tuberculosis* lipoprotein (TLR2/IL) [10]. Dysregulation of the vitamin D pathway may be influenced by VDBP and VDR gene polymorphisms that regulate the upstream vitamin D bioavailability and downstream transcriptional activity in response intracellular pathogens.

The genes controlling VDBP and VDR are highly polymorphic and have been associated with the dysregulation of the innate immune response [11]–[16]. Single-nucleotide polymorphisms (SNPs) D432E (GenBank rs7041) and T436K (GenBank rs4588) result in changes in the VDBP protein structure that produce the three most common variants of VDBP; Gc1f, Gc1s and Gc2. These variants differentially influence the bioavailability of VDBP and its binding affinity to 25-OHD_3_ and the induction of cathelicidin in monocytes [6], [11], [17]. Five VDR SNPs (restriction fragment length polymorphisms identified using the enzymes Fok1, Bsm1 (GenBank rs1544410), Apa1 (GenBank rs7975232), Taqα1 (GenBank rs731236), and Cdx2 (GenBank rs11568820)) result in differential gene transcription and are associated with changes in bone mineral density, calcium absorption, vitamin D-related disease conditions, metabolic disorders, and susceptibility to infectious diseases [16], [18]–[21].

The purpose of this study was to; 1) determine the contribution of vitamin D from food sources and measure the serum concentrations of 25-OHD_3_ and VDBP; 2) detect the frequency of VDR and VDBP SNPs that are associated with dysregulation of the innate immune response, and 3) evaluate potential correlations between VDBP SNPs and VDBP serum concentrations. This analysis of the vitamin D pathway is part of a larger community participatory research partnership focused on the biologic and social determinants of health and illness, particularly infectious diseases such as tuberculosis, which is endemic and epidemic in the Dené community and in this region of Canada [22]–[24]. The investigation of the genetic and environmental conditions related to vitamin D and the innate immune response may contribute to a better understanding of the risk factors for infectious diseases.

## Results

The total number of study participants was 46 (21 males/25 females). The mean age (41.6±15.6 years) and body mass index (BMI) (30.7±6.3) did not differ significantly by sex. There were no significant correlations between age, gender or BMI in either season (data not shown). The participants were generally healthy. The most common chronic illness was hypertension (N = 7 (15%)); other conditions included gastroesophageal reflux, asthma and eczema. The mean number of chronic diseases per participant (0.6±1.0) was calculated by averaging the number of diseases across all participants to assess health. No participant was taking a medication that affected vitamin D absorption. Eleven participants self-reported a diagnosis of latent tuberculosis, and nine others self-reported having had active tuberculosis that was treated in the past.

### Dietary Vitamin D Assessment

The mean daily intake of vitamin D was 271.4 IU/day in winter and 298.3 IU/day in summer (Table 1). There was no significant difference in mean daily vitamin D intake within any of the sub-groups (season (winter/summer), sex (male/female), age (<40 years/≥40 years) or BMI (<25/≥25)). Fifty percent of the daily vitamin D came from milk (fluid and powder) in the winter and summer. Local fish provided 20% of the vitamin D in the winter and 28% in the summer (Figure S1). The mean vitamin D intake of participants who took vitamin D supplements was significantly higher than those who were not taking supplements (Table 1).

**Table 1:** Vitamin D intake, and serum concentrations of 25-OHD_3_ and VDBP for the study participants by demographic group and season (mean ± standard deviation).

	Vitamin D Intake (IU/day)			25-OHD_3_ concentration (nmol/L)		VDBP concentration (ug/ml)			
Variable (N = winter, summer)		Winter	Summer	Winter	Summer	Winter	Summer		
Total (N = 45, 46)		271.4±214.4	298.3±226.1	66.5±34.5	103.3±41.8∧	364.3±124.6	204.5±78.8∧		
Males (N = 20, 21)		285.0±222.3	311.3±233.8	63.5±26.0	100.9±44.5∧	375.7±158.1	204.2±94.2∧		
Females (N = 25, 25)		260.2±211.8	287.4±223.7	69.9±40.7	105.3±40.2∧	353.0±94.6	208.2±64.9∧		
<40 years old (N = 19, 20)		216.9±170.2	281.5±228.1	46.5±18.4	77.7±32.8∧	434.8±104.5	227.7±82.4∧		
≥40 years old (N = 26, 26)		276.0±236.9	320.0±228.3	82.1±36.3*	122.7±37.5*∧	310.7±114.9*	190.2±73.1*∧		
BMI (Kg/m^2^) <25 (N = 10, 10)		314.4±187.7	391.7±237.0	57.1±30.7	102.4±57.2	392.3±135.9	220.3±59.2∧		
BMI (Kg/m^2^) ≥25 (N = 34, 34)		259.9±223.9	277.9±223.7	67.0±31.8	103.5±37.2∧	354.8±123.5	202.3±83.8∧		
No vitamin D supplements (N = 39, 38)		231.0±198.2	257.0±216.1*	60.8±30.1	96.5±42.2∧	365.3±129.2	209.3±82.6		
Vitamin D supplements (<400 IU/day) (N = 6, 8)		534.1±135.3*	494.3±188.1	104.9±40.0*	133.9±25.1*∧	368.7±109.7	191.0±54.8		

∧difference between seasons p<0.005 (ANOVA).

* difference within sub-groups p<0.005 (ANOVA ).

### Serum Concentrations of Vitamin D

Mean serum concentrations of 25-OHD_3_ were significantly higher in the summer compared to the winter for the total study group and for each of the sub-groups (Table 1). Participants who were ≥40 years of age had higher mean serum concentrations than those <40 years of age in winter and summer.

Serum concentrations of 25-OHD_3_ were positively correlated with vitamin D intake in the winter (R = 0.4, p = 0.002) but not in the summer (R = 0.03, p = 0.25). Age also had a positive correlation with serum concentrations of 25-OHD_3_ in the winter (R = 0.75, p<0.005) and summer (R = 0.4, p<0.005). BMI was not correlated with serum concentrations of 25-OHD_3_ in either season. Overall, 61% and 30% of the study participants had serum concentrations <75 nmol/L in the winter and summer respectively. The mean concentrations of 25-OHD_3_ were significantly higher for those participants who took vitamin D supplements (≤400 IU/day) in both winter and summer compared to those who did not (Table 1).

The mean serum concentrations of VDBP were significantly lower in the summer compared to the winter for the total study group and for each of the sub-groups (Table 1). Participants who were ≥40 years of age had significantly lower mean serum concentrations of VDBP than those <40 years of age in both winter and summer (Table1).

There was no correlation between concentrations of VDBP and any of the traits in the sub-groups in the winter (sex (R = 0.09 p<0.5), age (R = 0.1 p<0.3) or BMI (R<0.006 p = 0.5)) or summer (sex (R = −0.04 p<0.7), age (R = 0.07 p<0.6) or BMI (R<0.03 p = 0.8)).

**Figure 1:**
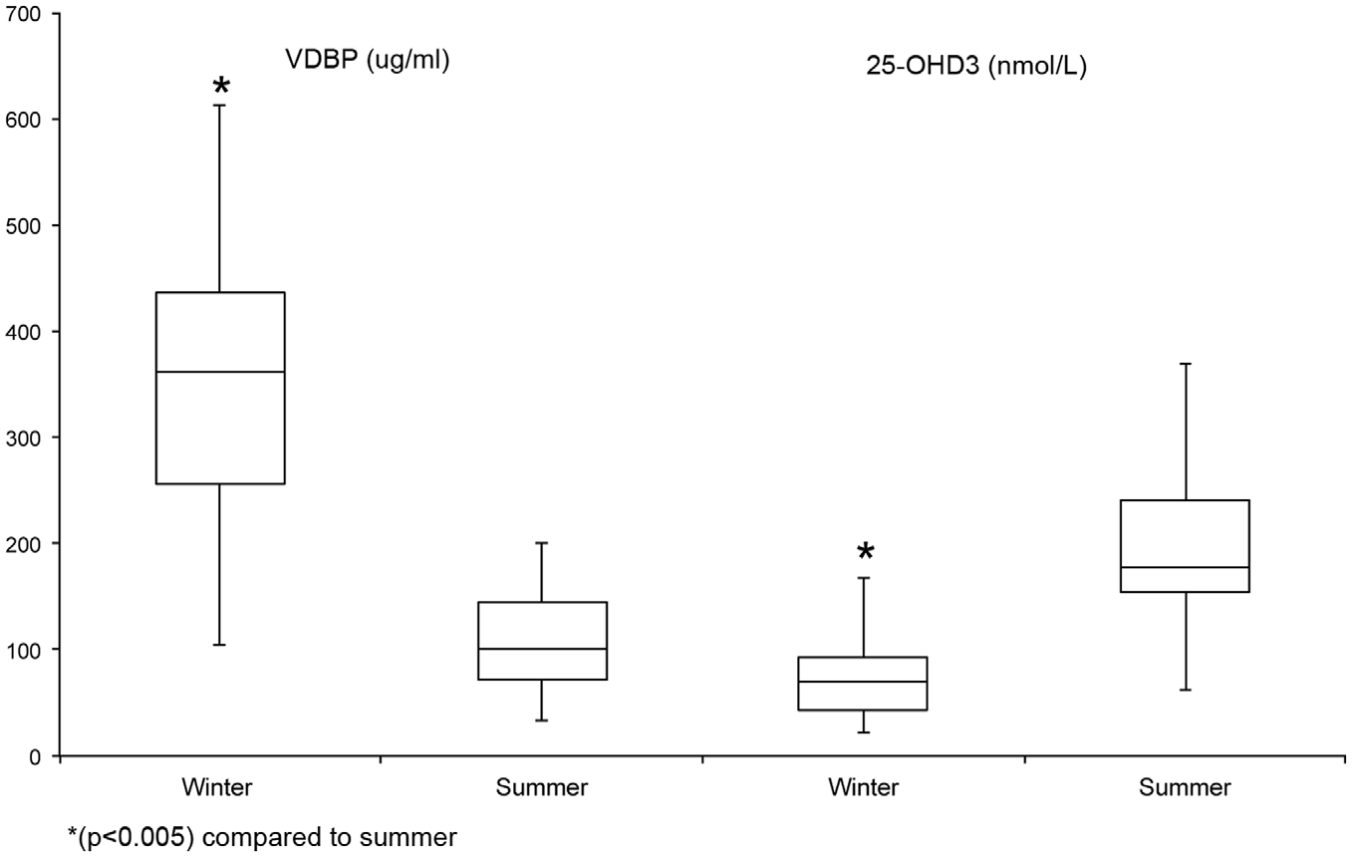
Seasonal comparison of the serum concentrations of VDBP (ug/ml) and 25-0HD_3_ (nmol/L). The mean serum concentration of VDBP was significantly lower in the summer. The mean serum concentration of 25-OHD_3_ was significantly higher in the summer. The concentrations of VDBP and 25-OHD_3_ were inversely correlated.

The mean 25-OHD_3_ concentrations were lower in the winter and higher in the summer and in contrast, VDBP concentrations were higher in the winter and lower in the summer for the study group (Figure 1). There were no correlations between serum concentrations of VDBP and 25-OHD_3_ in either season or in any demographic subgroup.

### VDBP and VDR Gene Polymorphisms

Genetic analysis of VDBP alleles was performed on all participants however the T436K SNP did not amplify in 6 samples. Allele and genotype frequencies for D432E and T436K were in Hardy-Weinberg Equilibrium (HWE). Table 2 summarizes the allele and genotype frequencies.

**Table 2:** Alleles and genotype frequencies for the vitamin D binding protein (D432E and T436K).

VDBP SNPs						N			%	
D432E (rs7041)										
	Alleles	G				65			71	
		T				27			29	
	Genotypes	G/G				20			45	
		G/T				21			48	
		T/T				3			7	
T436K (rs4588)										
	Alleles	C				77			91	
		A				8			9	
	Genotypes	C/C				28			70	
		C/A				12			30	
		A/A				0			0	
D432E+T436K										
	Diplotypes	Gc1f (T+C)							15	
		Gc1s (G+C)							75	
		Gc2 (T+A)							10	
	Haplotypes	Gc1f-1f				0			0	
		Gc1f-1s				11			27	
		Gc1s-1s				22			54	
		Gc1f-Gc2				1			2	
		Gc1s-Gc2				7			17	
		Gc2-Gc2				0			0	

One-way ANOVA was used to compare differences in VDBP concentrations by genotype. The D432E-G/G genotype had significantly higher VDBP concentrations in the winter compared to the G/T and T/T genotypes (p<0.005) but not in the summer (Table 3). No differences in VDBP concentrations were found between for the T436K genotypes (Table 3). No correlation was found between serum concentrations of 25-OHD_3,_ and VDBP genotypes (data not shown).

**Table 3:** Vitamin D binding protein serum concentrations (ug/ml) stratified by vitamin D binding protein genotypes (mean ± standard deviation).

	Winter	Summer
**VDBP Genotypes**		
D432E (rs7041)		
#G/G (N = 22)	469.4±72.85	246.4±72.61
T/G (N = 20)	277.2±66.40*	173.9±58.51
T/T (N = 3)	208.1±153.8*	143.9±120.6
T436K (rs4588)		
#C/C (N = 32)	381.3±131.4	217.1±80.50
C/A (N = 8)	290.8±63.6	165.0±57.2
A/A (N = 0)	0	0
**VDBP Haplotypes**		
D432E+T436K		
#Gc1f-1f (N = 1)	408.0±0	62.0±0
Gc1f-1s (N = 9)	380.6±170.8	193.9±75.0
Gc1s-1s (N = 23)	339.6±116.8	241.4±70.85
Gc1f-Gc2 (N = 2)	332.2±148.4	205.8±108.3
Gc1s-Gc2 (N = 6)	414.3±99.5	154.4±41.1
Gc2-Gc2 (N = 0)	0	0

#reference group.

* within season comparison p<0.005 (ANOVA).

The three most common VDBP diplotypes (Gc1s, Gc1f and Gc2) were all present in the Dené study group. The Gc2 and Gc1f have aspartic acid (GAT) at position 416 compared to a glutamic acid (GAG) in the Gc1s allele. Gc2-A allele encodes for lysine amino acid (AAG) at position 420 compared with the threonine (ACG) in Gc1f and Gc1s. The combination of glutamic acid and lysine is rarely seen in humans so only the three variants are possible. The Dené had a greater frequency of the Gc1 (Gc1f and Gc1s) alleles compared to the Gc-2 (Table 2). The Gc1 diplotype is characterized by high and intermediate binding affinity for 25-OHD_3_, whereas the Gc2 diplotype is associated with a low binding affinity [6].

The mean serum concentrations of VDBP were significantly higher for the D432E – G/G genotype compared to the G/T and T/T genotypes (Table 3). The mean concentrations of VDBP were compared by haplotype and no significant differences were found (Table 3).

The VDR gene has more than 470 polymorphisms but only a small number have been demonstrated to have functional effect [16], [25]–[34]. In our study the Apa1 and Taqα1genotypes were in HWE (Table 4). The Fok1, Bsm1 and Cdx-2 sites were not in HWE likely due to the small sample size or as a result of selective pressure on the alleles. The Dené had a high frequency of the Fok1-f SNP (82%), which results in an alteration of the start codon. The Fok1-f SNP has moderately lower VDR transcriptional activity than the Fok1-F SNP [7], [11], [19]. The Bsm1-b allele and the b/b genotype at the Bsm1 restriction site occurred most frequently (b-96% and b/b-89%) in the Dené cohort compared to the major allele and genotypes. This pattern of allele and genotype frequency was also noted for the Taqα1 and Cdx2 restriction sites (Taqα1-T allele - 96% and Taqα1-T/T genotype–89%; Cdx2–G allele–96%; Cdx2-G/G genotype-91%).

**Table 4:** Allele and genotype frequencies for the vitamin D receptor gene polymorphisms (Fok1, Bsm1, Apa1, Taqα1 and Cdx2).

VDR SNPs					N		%	
Fok1 (rs10735810)								
	Alleles		C (F)		13		18	
			T (f)		74		82	
	Genotypes*		C/C (F/F)		4		8	
			C/T (F/f)		9		20	
			T/T (f/f)		33		72	
Bsm1 (rs1544410)								
	Alleles		T (B)		4		4	
			C (b)		86		96	
	Genotypes		T/T (B/B)		1		2	
			T/C (B/b)		4		9	
			C/C (b/b)		41		89	
Apa1 (rs7975232)								
	Alleles		T (A)		29		32	
			G (a)		61		68	
	Genotypes*		T/T (A/A)		4		9	
			T/G (A/a)		22		48	
			G/G (a/a)		20		43	
Taqα1 (rs731236)								
	Alleles		T (T)		86		96	
			C (t)		4		4	
	Genotypes		T/T (T/T)		41		89	
			T/C (T/t)		5		11	
			C/C (t/t)		0		0	
Cdx2 (rs11568820)								
	Alleles		G		86		96	
			A		4		4	
	Genotypes*		G/G		41		91	
			G/A		4		9	
			A/A		0		0	

(*not in Hardy-Weinberg Equilibrium p<0.005).

No correlation was found between serum concentrations of 25-OHD_3_ or VDBP and the VDR gene polymorphisms (data not shown).

## Discussion

The interaction between diet, environment, and the genes that influence the vitamin D pathway impacts the functioning of a broad range of human tissue and cell types. Vitamin D’s effect on calcium absorption is critical for skeletal health. However, many cells, including macrophages, have the capacity to synthesize 1,25-OHD_3._ This process is integral to cellular development and health. Inadequate vitamin D intake both from UVB radiation or dietary sources can impact innate and adaptive immunity, and has been implicated in autoimmune disorders, chronic conditions and infectious diseases.

### Study Limitations

The use of culturally respectful research methodologies and knowledge translation processes in partnership with the people of this Dené First Nation, has yielded results that the community has indicated, hold legitimacy and meaning. However, the ability to generalize the study findings (external validity) to the general Dené population and to other First Nation communities is potentially limited due the small number of those recruited, and the method of recruitment. The sample size of the study group was small and the serum concentrations of 25-OHD_3_ and VDBP were highly variable. Other limitations include the possibility that the diet and sunlight exposure of those who chose to participate in the study differed from those who did not participate. In terms of the internal validity of the study, the use of a food questionnaire to calculate nutrient intake poses a challenge. Recognizing this, the questionnaire used in this study was field-tested and adapted to the community and their culture in an effort to maximize accuracy and reproducibility [24]. Future studies that include a larger sample will help to clarify the extent of seasonal and individual variability in the Dené population.

### Dietary Assessment

Recognizing these limitations, the results of this study demonstrate that optimal vitamin D intake from diet (with or without taking ≤400 IU/day of vitamin D) was not achieved by the participants in this study; 89% did not meet the 2010 Institute of Medicine’s recommended daily dietary intake of 600 IU/day of vitamin D [37]. Vitamin D intake was higher among the older study participants. Similar results were found for rural Aboriginal women over 50 year olds. This group had a mean intake that was higher than 25–50 year olds in the same study [35]. Kuhnlein found that the traditional diet of the Dené people of Canada’s Northwest Territory is being replaced by market foods, particularly among the younger age groups [36]. Increasing reliance on expensive market foods is occurring in most northern aboriginal communities. The causes include the increasing expense associated with hunting and fishing, environmental degradation, climate change, and loss of traditional knowledge [37]–[39].

### Serum Concentrations of 25-OHD_3_

Despite the consistently low dietary intake of vitamin D in winter and summer, the mean serum concentrations of 25-OHD_3_ varied between seasons. Taking low doses (≤400 IU/day) of vitamin D supplements significantly increased serum concentrations of 25-OHD_3_ compared to participants who did not take supplements. However, the majority of Dené participants (61%), including those taking vitamin supplementations (≤400 IU/day), did not meet current recommended serum concentration of 25-OHD_3_ (≥75 nmol/L) during the winter [40]–[42]. Summer concentrations of 25OHD_3_ were ≥75 nmol/L for 70% of the participants likely due to sunlight exposure.

Serum concentrations of 25-OHD_3_ and VDBP were correlated with age. Compared to the <40 year olds, the ≥40 year olds had lower mean concentrations of VDBP and higher mean concentrations of 25-OHD_3_. There was also a positive correlation between increasing age and serum concentrations of 25-OHD_3_. Most studies report decreased vitamin D concentrations in older individuals and attribute this to a more restricted diet, less physical activity and time spent outdoors [43], [44]. Higher serum concentrations of vitamin D in the older individuals in the Dené community might be attributed to the older adults spending more time outdoors than those participants who were <40 years olds or increased consumption of local fish which is high in vitamin D [45].

In support of the importance of UVB as a vitamin D source, the serum 25-OHD_3_ concentrations were correlated with dietary vitamin D in the winter but not in the summer. This finding is in contrast to other studies that found a significant correlation between dietary intake of vitamin D and serum concentrations of 25-OHD_3_ in winter and summer/fall [35], [46]–[49]. This discrepancy may be related to a number of factors, including possible flaws in the food frequency questionnaire, the small sample size or the relative unavailability of vitamin D containing foods. More likely it is related to fact that the major source of vitamin D is linked to an individual’s sunlight exposure making dietary intake a relatively minor source [50].

The findings of low winter and summer vitamin D dietary intake among participants in this study, and low winter serum concentrations of 25-OHD_3_ were in alignment with previous studies among circumpolar and Canadian indigenous populations [3], [4], [40], [41], [46]–[48], [51]. However, the serum concentrations of 25-OHD_3_ in this study were higher than reported for other Aboriginal groups (41.8±14.5 nmol/L) [35]. The continued consumption of traditional food sources (fish) and increased UVB exposure in the summer by participants in this study may explain the higher 25-OHD_3_ concentrations. While vitamin D deficiency, is clearly not an issue for the majority of study participants, in the summer, vitamin D insufficiency in the winter may play a role in dysregulation of the immune response to the current situation of endemic tuberculosis.

### Serum Concentrations of VDBP

In this study serum concentrations of VDBP were within the normal range (300–600 µg/ml) in the winter but below normal levels in the summer [6]. Serum concentrations of VDBP are highly variable within and between studies, and cell and tissue trauma caused by liver diseases, nephrotic syndrome and malnutrition can result in low VDBP concentrations [6]. To our knowledge there are only a few studies that report seasonal serum VDBP concentrations. Seasonal differences were found in VDBP concentrations in breast-fed infants but not in their mothers [49]. Although summer is short in the north, the increased availability of 25-OHD_3_ (as evidenced by the higher serum concentrations in our study group) through UVB exposure may have an effect on the concentrations of VDBP. One of the many functions of VDBP is to bind with 25-OHD_3_ and 1,25-OHD_3_ and transport it to target cells. VDBP also binds and transports fatty acids, scavenges actin and has a role in macrophage activation [6], [17], [52]. VDBP is typically present in high concentrations (5×10^−6^ M) and has a short half-life (2.5 days) compared to 25-OHD_3,_ which has a half-life of 12 days and plasma concentrations of 5×10^−8^M.

### VDBP and VDR Gene Polymorphisms and their Functional Effects

VDBP gene polymorphisms have been shown to affect the serum concentrations of VDBP and its avidity to 25-OHD_3_. The D432E–G allele and the Gc1 diplotype are associated with higher concentrations of VDBP, intermediate to high binding affinity, and slower metabolism of 25-OHD_3_. For our study group, given the low UVB exposure in the winter there would be an advantage to having high concentrations of VDBP with high binding affinity to maximize the limited availability of vitamin D. The Dené had a high frequency of the VDBP D432E– G allele and the G/G genotype was associated with higher concentrations of VDBP in the winter but not in the summer. In contrast to other studies there was no association between VDBP genotypes and concentrations of 25-OHD_3_ [17].

Population genetic studies have shown an association between Gc1f, Gc1s and Gc2 variants of VDBP, geographic distribution of populations, and insulin resistance. Among the Dogrib Dené the Gc1f genotype was associated with lower measures of fasting insulin than those with the Gc2 genotype [6], [41], [53]. The Gc1f genotype has also been shown to be protective against Type II diabetes [54]. In another study the lower affinity Gc2 genotype was correlated with higher induction of the antibacterial peptide LL-37 by 25-OHD_3_ [10]. The homozygous Gc1f genotype is a significant risk factor for the development of chronic obstructive pulmonary disease (COPD) and for carriers of the Gc1f variant the age related decline of the Forced Expiratory Volume (FEV_1_) was significantly higher (6). It is possible that selection for the Gc2 genotype occurred in populations exposed to recurrent intra-cellular infections, which might account for its higher frequency among European decent groups [55]–[57].

The VDR genotypes found in high frequency among Dené participants have been associated with decreased macrophage response to pathogens, and a number of chronic, and infectious diseases, cancer, osteoporosis, autoimmune conditions, multiple sclerosis and tuberculosis [5], [16], [30]–[33], [58]–[61]. The Dené group in this study did not differ significantly from previously published results for a separate Dené cohort from the same community [23].

The VDR Fok1 restriction site defines a SNP in the first of two potential translation initiation start sites for the VDR mRNA. Two protein variants can exist corresponding to the two available start sites: the longer VDR, encoded by the alternate allele form (ATG) (designated f), is three amino acids longer and 1.7 times less efficient than the common allele form (ACG) (designated F) [27]. This has functional consequences for the intra-cellular activity whereby the amino acid structure created by the Fok1-f allele will reduce the transcriptional activity and the production of antimicrobial cathelicidin [58]. The Cdx2 SNP has an important role in the transcriptional activity of VDR in the small intestine, calcium absorption, and bone mineral density [27]. The VDR promoters that have the Cdx2-G allele have 70% less transcriptional activity than the A allele resulting in poorer calcium absorption.

The VDR restriction sites Bsm1, Apa1 and Taqα1 have not been demonstrated to have an effect on gene expression but they may be linked to functional SNPs [25], [26]. There are however studies that have found associations between these SNPs and infectious diseases [30], [59], [60]. In a recent study in India, individuals with Bsm1-b/b, Fok1-f/f, Taqα1-t/t genotypes were at higher risk of developing multi-drug resistant tuberculosis and/or smear positive disease than heterozygotes or homozygous dominant individuals [62]. The high frequency of the Fok1-f, Bsm1-b, Apa1-a and Taqα1-T alleles in the Dené population may increase their risk of tuberculosis infection or disease. The combination of 25-OHD_3_ deficiencies, the high frequency of the Fok1-f/f genotype, and the Taqα1-T/T or T/t genotype, were strongly associated with tuberculosis in an Asian population [30]. Children with the Fok1-f/f genotype were also at increased risk of acute lower respiratory infection and in another study, women with this genotype had an increased risk of breast cancer [25], [31], [32]. Although the Fok1-f allele is associated with a number of adverse outcomes, there may be selective pressures that favour this allele for functions related to tissue, bone and/or muscle. Despite the inconsistencies that occur between studies with regards to VDR SNPs and bone mineral density, there does appear to be an association between Bsm1, Fok1 and bone development. The Fok1-f allele was associated with muscle strength in homozygote females and the Bsm1-b allele with increased bone mineral density [63]. In other studies the Bsm1-b and Fok1-f alleles have both been associated with low bone mineral density [27], [28].

### Summary

Among the Dené participants of this study, low winter serum levels of 25-OHD_3_ were likely related to dietary deficiency and limited exposure to sunlight-induced cutaneous production of vitamin D. In this context, the high frequency of VDBP genotypes associated with avid binding affinity, and VDR alleles associated with reduced transcriptional activity, may adversely affect the diverse functions of this vitamin, including innate immune responses to pathogens. The observed VDBP and VDR allele and genotype frequencies in the study group may reflect population based selective pressures for the so called “classic vitamin D pathways” (calcium absorption, bone mineralization and remodelling, anti-inflammatory regulation and rapid tissue repair). In northern First Nation communities where infectious diseases such as tuberculosis are endemic, and high quality nutritious foods are difficult to access, a vitamin D pathway that strongly down-regulates a Th1 immune response may have serious health implications [64], [65].

Identification of nutrient deficiencies, and detection of functional gene polymorphisms associated with infectious and chronic disease, may provide guidance for future prevention and treatment programs. To-date, studies on vitamin D and innate immune regulation have occurred only in non-aboriginal groups with one recent exception [10]. In vitro studies of vitamin D metabolism and innate immunity in aboriginal groups are crucial for clarifying the regulatory pathways and function of vitamin D in this population. This may pave the way for in vivo supplementation trials.

## Materials and Methods

### Ethics Statement

Canadian Aboriginal research principles of ownership, control, access and possession (OCAP) were followed (The First Nations Principles of OCAP. Ottawa: First Nations Governance Centre; 2010. Available at: http://www.fnigc.ca/node/2). The University of Manitoba Health Research Ethics Board and the community’s Chief and Council approved the study. Study participants were 18 years of age or older and provided informed written consent.

### Study Participants

Northlands Denésuline First Nation (Dené) members were recruited at Lac Brochet, located at 58° latitude in northern Manitoba, Canada. The Dené community in this study is remote and accessible only by air and winter road. The Dené are part of the larger Na-Dené (Athapaskan) language family, which includes the Alaskan Gwich’in and the American Apache and Navajo. The Dené have a long history of reliance on migratory caribou and fish as primary food sources. However, their food security is currently challenged by environmental, social, economic, and political pressure [37]–[39].

This community is comprised of 145 census families which include 605 individuals (350 of whom are ≤19 years of age) who self-identified as Dené [66]. Community consultation determined that convenience sampling, rather than random sampling, was the only methodology considered acceptable. Participants were eligible for inclusion if they were 18 years of age or older, able and willing to give informed consent, self-identified as Dené, able to commit to participation for the duration of the study and committed to avoid taking vitamin D supplements >400 IU/day. Individuals taking a low dose of vitamin D (≤400 IU/day) were allowed to participate in the study. In this way it was hoped that the study cohort might more accurately reflect the general community population (Figure S2). Supplementary vitamin D intake was added to the calculation of dietary intake. Forty-six participants (representing approximately 14% of the adult population) completed the study.

Data collected in winter (February-March) and late summer/fall (September-October) of 2010, included age, sex, body mass index (BMI), medications, chronic health conditions and self-identified ethnicity. A food frequency questionnaire (FFQ) from Wu [67] was modified to include available vitamin D containing market foods (i.e. milk, margarine, etc.) and traditional foods (i.e. local fish caribou fat, meat and organs). The frequency of vitamin D containing foods and portion sizes were assessed using the vitamin D values from the Canadian Nutrient File [45]. The questionnaire was field-tested and administered at winter and summer time points. Participants were asked to recall food consumption patterns for the previous month.

### Analysis of 25-OHD_3_ and VDBP Serum Concentrations by ELISA

Venous blood samples were collected at the winter and summer visits in 2010.

Serum concentrations of 25-OHD_3_ and VDBP were evaluated by ELISA (Immunodiagnostic Systems, Inc. Scottsdale, AZ, USA and R&D Systems, Minneapolis, MN, USA) [68]. For the 25-OHD_3_ ELISA intra- and inter-plate coefficients of variation were 2.2±2.9% and 5.7±1.4%. For the VDBP ELISA intra- and inter-plate coefficients of variation were 3.3±2.4% and 8.6±6.2%. Vitamin D sufficiency in this study was based on serum concentrations of 25-OHD_3_≥75 nmol/L [40]–[42].

### SNP Analysis

Genomic DNA was manually extracted from buffy coats using the QIAamp DNA Blood Mini Kit (Qiagen Inc. Toronto, ON). The following PCR conditions were used for VDBP amplification in a 25 µl reaction: 3.5 µl extracted DNA; 2.5 µl 10X PCR Buffer; 1.0 µl 50 mM MgSO4; 0.2 µl 10 mM dNTP mix; 16.2 µl reagent grade water; 0.1 µl Platinum® Taq (Invitrogen Life Technologies Corp. Burlington, ON); 0.5 µl each of the forward primer and reverse primers [52]. PCR conditions were 95°C for 15 min followed by 35 cycles of 94°C for 20s, 58°C for 20s, and 72°C for 20s with a 1sec increment for each subsequent cycle, and one cycle at 72°C for 10 min [25]. PCR amplification of VDR SNPs Bsm1 (T/C), Apa1 (G/T), Taqα1 (C/T), Fok1(T/C), and Cdx-2 (G/A), was performed using published protocols and primers [25]–[27]. Analysis of the VDR SNPs at the restriction sites Bsm1 (B/b (T/C)), Apa1(A/a (T/G)), Taqα1(T/t (T/C)), Fok1 (F/f (C/T)), and Cdx-2 (G/A) and VDBP SNPs at D432E (G/T) and T436K (C/A) were detected using RFLP and were visualized with ethidium bromide staining and ultraviolet illumination [25]–[27]. Allele counting was conducted manually.

### Statistical Analysis

Statistical analysis was preformed using AnalystSoft, StatPlus:mac - statistical analysis program for Mac OS. Version 2009. Comparison of mean concentrations of 25-OHD_3_ and VDBP were evaluated between demographic groups (sex, age, BMI) and seasons (winter/summer) using Pearson’s Correlation or Student’s-T Test on the transformed data.

The Shapiro-Wilk test was used to assess the null hypothesis that the sampled concentrations of vitamin D (25-OHD_3_) and VDBP were normally distributed population. The winter concentrations of 25-OHD_3_ were skewed and this data was log transformed for multiple regression analysis. Differences in mean concentrations of 25-OHD_3_ and VDBP were evaluated by season, age, sex, BMI, and vitamin D intake were evaluated for the main effects using ANOVA. Multiple regression was used to test correlations between the vitamin D intake, season, age, sex, BMI (independent variables), and concentrations of 25-OHD_3_ (dependent variable). VDBP concentrations were dependent variables in a multiple regression using season, age, sex, BMI were the independent variables.

VDBP and VDR allele and genotype frequencies were evaluated for deviations for Hardy-Weinberg Equilibrium (HWE) using a chi-square test. Associations between VDBP allele, genotypes, and serum concentrations of 25-OHD_3_ and VDBP, were analyzed using multiple regressions in separate analyses for winter and summer.

Seasonal 25-OHD_3_ and VDBP concentrations were evaluated separately against T436K and D432E alleles using multiple linear regressions controlling for BMI, age and sex. VDBP concentrations were used as the dependent variable and T436K and D432E alleles 1 and 2 were independent variables.

P values <0.05 were considered statistically significant unless otherwise stated.

## Supporting Information

Figure S1 **Relative (%) contribution of traditional and market foods to daily vitamin D intake (IU/day) by season.** Fifty percent of the dietary intake of vitamin D came from milk (fluid and powdered). Local fish obtained from the lake provided 20% in the winter and 28% in the summer of the daily vitamin D. Margarine and eggs were also source of vitamin D. Caribou was not a major source of vitamin D however values for some animal parts that were consumed were unknown (i.e. fat, blood, liver).
(TIF)

Figure S2 **Enrolment of study participants.** One-hundred and five of 330 adults in the community were screened for the study [68]. Exclusion criteria included the use of vitamin D supplements >600 IU/day for three months prior to study on-set, clinical evidence of infection at the time of enrolment, first-degree kinship with an individual already enrolled, and immunosuppressive medical condition or use of immunosuppressive medication, including systemic steroids. Fifty-four of the 105 people screened met the study criteria and were enrolled. During the course of the one-year study 2 individuals moved away permanently from the community, 2 were absent from the community during one of the two test periods, 3 individuals withdrew for personal reasons, and 1 person developed a serious inter-current illness precluding further study participation.
(TIF)
