# Supplementary figures and images for: Vitamin D in a Northern Canadian First Nation Population: Dietary Intake, Serum Concentrations and Functional Gene Polymorphisms
Source: PLoS One. 2012 Nov 21;7(11):e49872. doi: 10.1371/journal.pone.0049872 (PMC3503822; doi:10.1371/journal.pone.0049872)

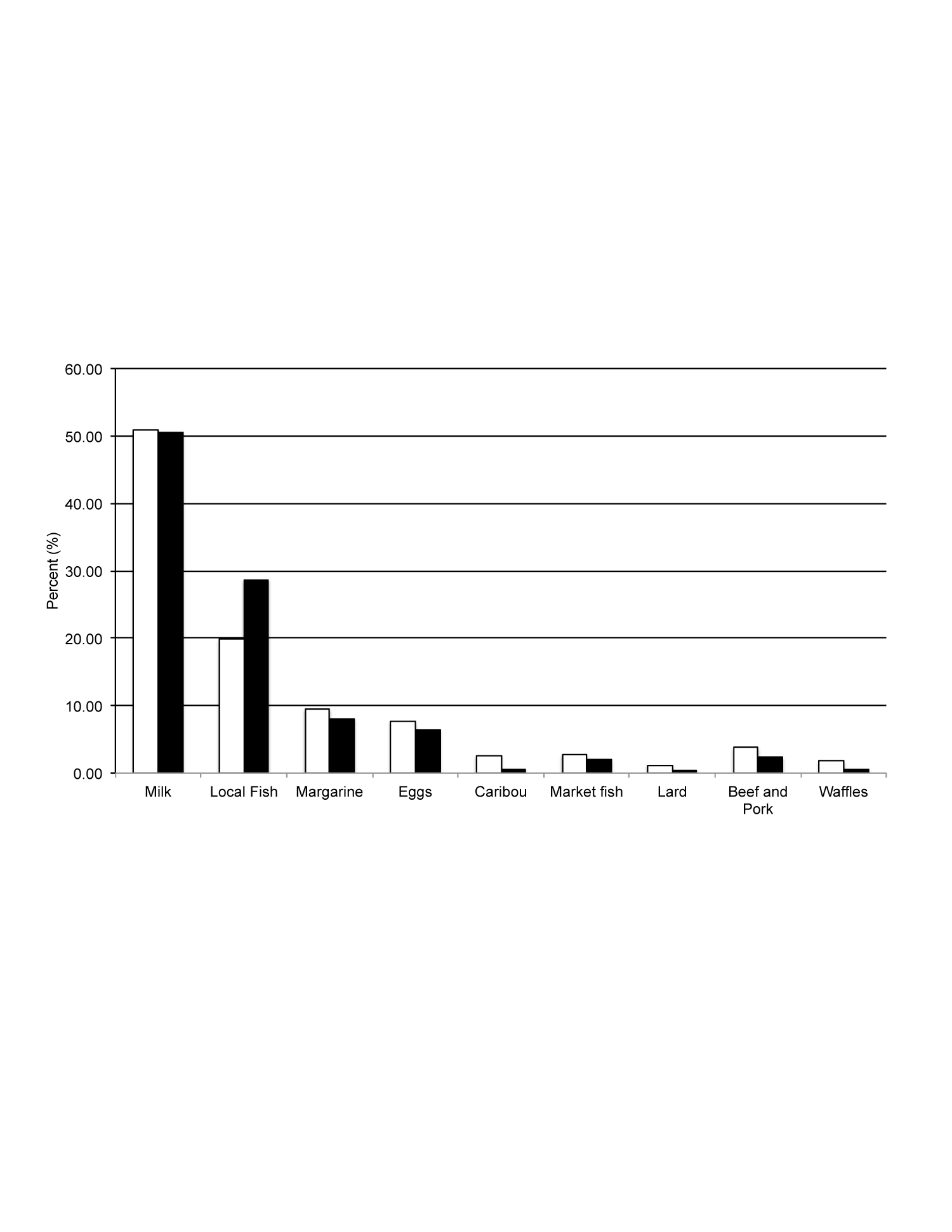

Supplement: Figure S1 — Relative (%) contribution of traditional and market foods to daily vitamin D intake (IU/day) by season. Fifty percent of the dietary intake of vitamin D came from milk (fluid and powdered). Local fish obtained from the lake provided 20% in the winter and 28% in the summer of the daily vitamin D. Margarine and eggs were also source of vitamin D. Caribou was not a major source of vitamin D however values for some animal parts that were consumed were unknown (i.e. fat, blood, liver). (TIF) [file pone.0049872.s001.tif]

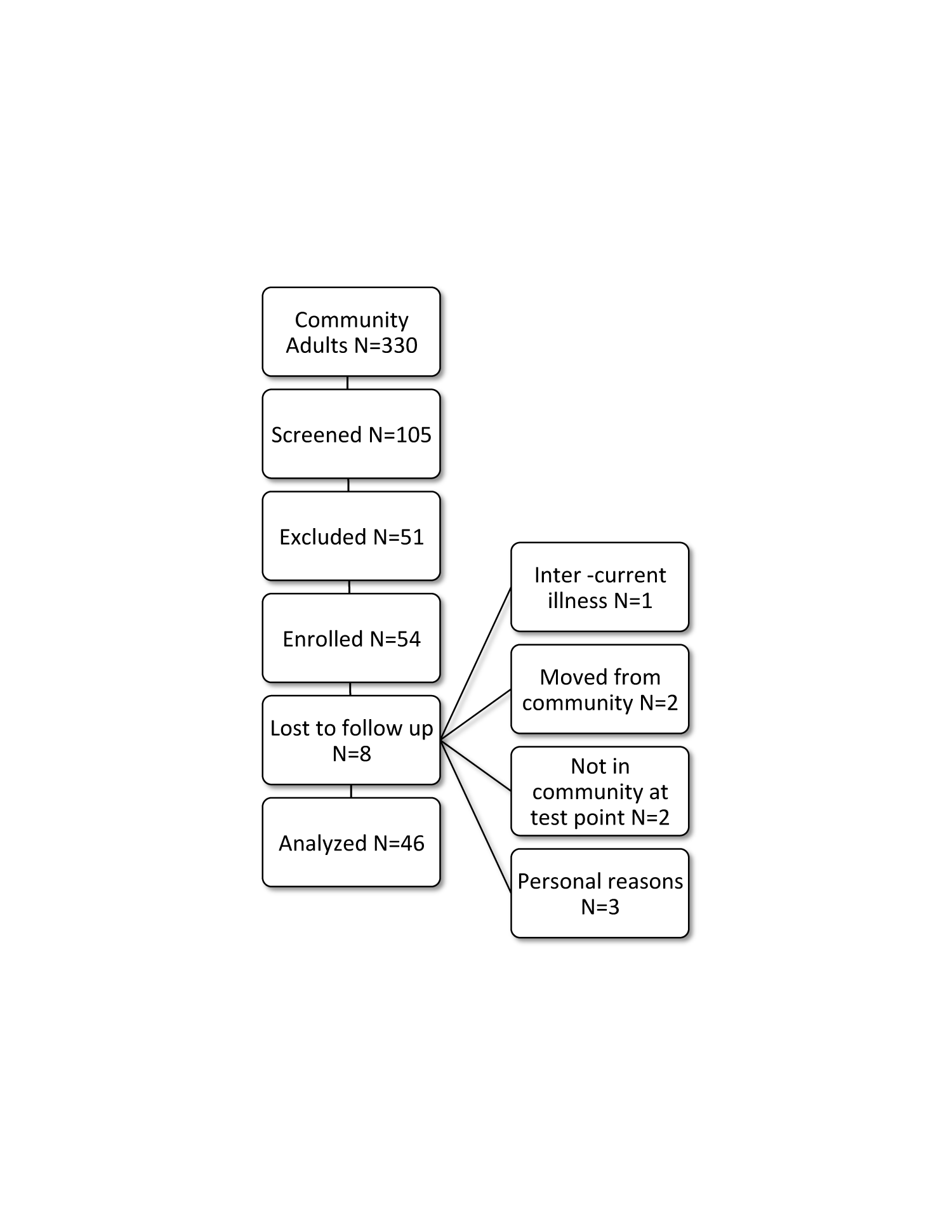

Supplement: Figure S2 — Enrolment of study participants. One-hundred and five of 330 adults in the community were screened for the study [68]. Exclusion criteria included the use of vitamin D supplements >600 IU/day for three months prior to study on-set, clinical evidence of infection at the time of enrolment, first-degree kinship with an individual already enrolled, and immunosuppressive medical condition or use of immunosuppressive medication, including systemic steroids. Fifty-four of the 105 people screened met the study criteria and were enrolled. During the course of the one-year study 2 individuals moved away permanently from the community, 2 were absent from the community during one of the two test periods, 3 individuals withdrew for personal reasons, and 1 person developed a serious inter-current illness precluding further study participation. (TIF) [file pone.0049872.s002.tif]
